# Feeding Practices, Prenatal Oral Health Information, and Early Childhood Caries in Toddlers Born Before and During the COVID-19 Pandemic: A Cross-Sectional Study from Hungary

**DOI:** 10.3390/dj14020101

**Published:** 2026-02-11

**Authors:** Andrea Radacsi, Krisztian Katona, Timea Dergez, Alexandra Jurasek, Marcell Herlicska, Istvan Somoskovi, Ildiko Szanto, Balazs Sandor

**Affiliations:** 1 Department of Dentistry, Oral and Maxillofacial Surgery, Medical School, University of Pecs, Tüzér Utca 1, 7623 Pécs, Hungary; radacsi.andrea@pte.hu (A.R.); katona.krisztian2@pte.hu (K.K.); jurasek.alexandra@pte.hu (A.J.); marcellherlicska@gmail.com (M.H.); somoskovi.istvan@pte.hu (I.S.); szanto.ildiko@pte.hu (I.S.); sandor.balazs@pte.hu (B.S.); 2 Department of Medical Statistics and Informatics, Institute of Bioanalysis, Medical School, University of Pecs, Honvéd Utca 1, 7624 Pécs, Hungary

**Keywords:** early childhood caries, COVID-19, caries prevalence, feeding pattern, dental awareness

## Abstract

**Objectives:** Early childhood caries (ECC) remains a significant global health issue. COVID-19 disrupted protective factors such as in-office parental support and routine dental screenings. This study investigates caries prevalence and severity in Hungarian toddlers (<3 years) born during the lockdown, compared with pre-pandemic data from 2019. **Methods:** A serial cross-sectional study was conducted through nursery-based dental screenings and a parental questionnaire. Key indices (caries prevalence; average number of decayed, missing, and filled tooth—dmf-t index; Significant Caries Index—SiC-index; and Restorative Index—RI) were calculated and analyzed in relation to parental education and knowledge of caries etiology. The results were compared to 2019 data. **Results:** A total of 636 children were examined; 274 (mean age: 29.37 ± 4.56 months) were part of the 2024 cohort. Caries prevalence decreased from 15.46% in 2019 to 13.87%. The mean dmf-t index also declined significantly (0.685 ± 2.20 vs. 0.383 ± 1.29; *p* = 0.025). Initial feeding practices, such as exclusive breastfeeding, were positively linked to later liquid intake habits (*p* < 0.01). Prenatal information did not affect caries rates or cariogenic liquid consumption. While parental education level was significantly related to caries prevalence and feeding practices in 2019, its influence was less marked in the 2024 cohort. **Conclusions:** ECC prevalence declined slightly among children born during the pandemic but remains high. Prenatal education did not promote healthier feeding-related oral health behaviors or outcomes. The reduced impact of parental education post-lockdown may suggest that heightened general health awareness during the pandemic lessened education-based disparities.

## 1. Introduction

In children under the age of 72 months, the presence of cavitated or non-cavitated caries on any tooth surface in the primary dentition, including filled or missing primary teeth due to caries, is classified as early childhood caries (ECC) [1,2]. ECC represents a serious health issue in both low-income and high-income countries [3]. Although ECC is a preventable disease, an estimate of more than 573 million children worldwide are still affected [4], and the vast majority remain untreated [5]. In the literature, caries occurring in children under the age of 3 is often referred to as severe early childhood caries (S-ECC) [6] (Table 1). The etiology and progression of ECC are significantly influenced by a diet rich in refined carbohydrates and insufficient oral hygiene; however, being a multifactorial disease, its etiological factors are not restricted to these. Microbiological, socioeconomic, and genetic backgrounds, as well as the parental (guardian’s) knowledge regarding how to prevent oral diseases, are critical factors in dental health and the development of ECC [7,8]. Educating and preparing parents to maintain their children’s oral health is the responsibility of healthcare professionals, including pediatricians, nurses, home visitors, and dentists conducting prenatal dental screenings [1].

In Hungary, according to current regulations (26/1997. [IX.3.] NM Decree on School 58 Health Care), regular dental screenings for children begin at age three within the framework of educational institutions: following children during their years in kindergartens and primary/secondary schools until adulthood [10]. This means that children under three are “invisible” to the dental healthcare system in our country, leaving no opportunity for early dentist–parent communication to support oral health or for early identification and treatment of pathological changes [6]. In contrast, the American Academy of Pediatric Dentistry (AAPD) emphasizes the importance of very early prevention and continuous dental care tailored to the child’s individual needs, recommending the first dental examination between the eruption of the first primary tooth and the child’s first birthday. This recommendation is consistent with the IAPD Bangkok Declaration on the prevention of ECC [5,9]. Dental treatment of decayed teeth in young children, particularly under the age of three, is time-consuming and challenging. Definitive or, in fact, any form of treatment is often not feasible in outpatient settings, and in some cases, general anesthesia is necessary [11]. While this option is available for mentally disabled patients in our country, it is not universally covered by health insurance for healthy patients, unlike in many European countries [12], underscoring the critical need for effective early prevention. Since April 2014, the Division of Pediatric Dentistry at the Department of Dentistry, Oral and Maxillofacial Surgery, Clinical Center of the University of Pécs, has been continuously providing comprehensive treatment under general anesthesia (within the framework of day care surgery) for such patients and ensuring their long-term care.

Studies examining the indirect effects of the COVID-19 pandemic have consistently reported worsening general health behaviors, including reduced physical activity, increased snacking and unhealthy dietary patterns, and declines in mental well-being, such as higher levels of anxiety and depression [13]. An international overview focusing on children and adolescents reported similar adverse lifestyle changes, with disruptions in physical activity, diet, and psychosocial well-being observed across multiple countries [14]. Nevertheless, some studies also indicate positive shifts in health awareness and preventive orientations, with certain populations reporting increased health consciousness and greater attention to healthier nutrition and self-care practices during the pandemic period [15,16].

Although oral hygiene is central to ECC prevention, this study focuses on early feeding behaviors and prenatal oral health-related information, as these factors are established during the first years of life, before regular toothbrushing routines are consistently implemented. Longitudinal evidence indicates that feeding practices in the first two years of life, including early sugar exposure and bottle use, are associated with ECC development, underscoring the role of early-life dietary determinants prior to the establishment of stable oral hygiene habits [17].

In this context, the present study sought to assess the dental health of children under three years of age attending nurseries in Pécs and compare the results with those of the previous study conducted before the COVID-19 pandemic. This age group was chosen because children under three years of age are not covered by Hungary’s mandatory kindergarten attendance requirements and are therefore not included in age-appropriate, regularly scheduled dental screenings, unlike older children attending kindergarten or school.

Reducing sugar consumption is a key element in dental prevention. A study by Paglia et al. demonstrated that diet, particularly the consumption of refined carbohydrates and their method of intake, is the main cariological factor in early childhood [18]. Therefore, an aim of the present study was to explore whether parents received oral health advice from healthcare professionals during pregnancy and to assess how such guidance may have influenced their child’s dietary habits and caries experience.

Preventing early childhood caries requires effective communication by healthcare professionals (dentists, dental assistants, etc.) to educate parents/guardians about this condition. However, with the outbreak of COVID-19, elective medical interventions and all screenings—including dental screening during pregnancy—were shut down. Our hypothesis was that the COVID-19 pandemic adversely affected the oral health of children born during this period, resulting in a higher prevalence and greater intensity of dental caries in this population.

## 2. Materials and Methods

We conducted a serial cross-sectional comparative study approved by the University of Pécs Ethics Committee (Approval No. PTE/75208/2018; Approval date: 10 October 2018), which covered participant recruitment, data collection, and statistical analysis. Epidemiological data on the prevalence of caries among children under three years of age in Hungary have been available only since 2021, based on data collected by our research team in 2019 [6]. The present study was designed to compare epidemiological patterns and parental knowledge in children born during the COVID-19 lockdown with pre-pandemic data, in the context of reduced access to routine screening and preventive guidance during pregnancy.

This study was carried out in 12 nurseries in Pécs, Hungary, including all nurseries maintained by the Municipality of Pécs and the nursery operated by the Faculty of Medicine, University of Pécs. Dental screenings were conducted in two phases, in 2019 and 2024. No institutions declined participation.

No formal prior sample size calculation was performed, as this study was exploratory and population-based, aiming to include all eligible children attending participating nurseries during the screening periods. The final sample size was therefore determined by institutional attendance and parental consent rather than statistical power estimation. The inclusion criteria were children attending the participating nurseries who were 36 months of age or younger at the time of examination and for whom written informed consent was obtained from a parent or legal guardian. Children older than 36 months were excluded. Although a total of 750 children were registered in the participating nurseries, several were absent or noncooperative on the initial screening days due to illness or other reasons, and in some cases, written parental or guardian consent was not available. Consequently, the nurseries were revisited to include children who were absent/noncooperative at the first visit; however, some children ultimately remained unexamined. Because no data were available on children whose parents declined participation or who remained unexamined, selection bias cannot be excluded.

Prior to participation, parents or guardians provided written informed consent. The nurseries did not grant information on the proportion of parents or guardians who declined participation.

**Figure 1:**
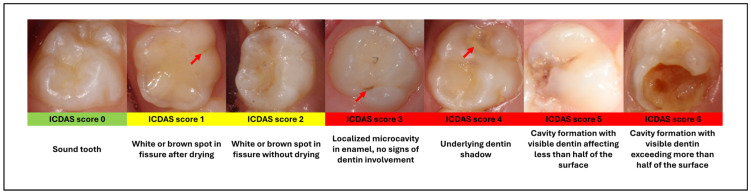
Caries diagnosis with the ICDAS scoring system. (ICDAS: International Caries Detection and Assessment System). Red arrows indicate the carious lesion. The figure was edited from our previous publication [6].

Screening protocol: Identical screening protocols were used in the 2019 and 2024 study phases, including examiners, training, and examination procedures. The screening was conducted in nurseries after scheduling appointments. It was performed before breakfast, following the children’s at-home oral care. For the examination, disposable dental mirrors and light sources such as headlamps or smartphones were used. Teeth were evaluated using the standardized and internationally recognized International Caries Detection and Assessment System (ICDAS), also used in everyday practice at our university clinic. Two pediatric dentists with 6–10 years of experience conducted the examinations, with dental assistants/nurses manually recording the results on standardized status sheets. To ensure examiner reliability, the dentists completed an online e-learning self-assessment program prior to the study (International Caries Classification and Management System; https://www.iccms-web.com/content/resources/elearning (accessed on 18 November 2018)), and no formal kappa calculation was performed. Examiners were trained in the full ICDAS scoring system, however lesion severity was not analyzed in detail during the study, and caries status was dichotomized:

- Carious: lesions scored ICDAS ≥ 3 (Figure 1).
- Sound teeth and non-cavitated lesions: teeth without cavitated lesions; incipient (reversible) lesions that scored ICDAS 1–2 were not recorded due to constraints of the examination environment [19].

Individual results were compiled on personalized forms distributed to parents by nursery personnel.

**Figure 2:**
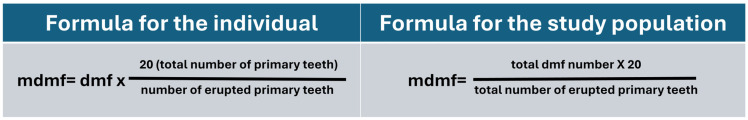
The method for calculating the proposed modified dmf-t index. (dmf: number of decayed, missing due to caries, and filled teeth; mdmf: modified dmf).

Index calculations: dmf-t index (number of decayed, missing due to caries, and missing teeth) [20], Significant Caries Index (SiC) [21], and Restorative Index (RI, f/d+f) were calculated for the study population. In our previous study (first phase), we proposed the use of a modified dmf-t index (mdmf-t) adjusted for the number of erupted primary teeth to account for the incomplete eruption commonly observed in young children. As the conventional dmf-t index assumes a fully erupted primary dentition of 20 teeth, its direct application in populations with partially erupted dentitions may lead to an underestimation of caries intensity. The modified index scales the observed dmf-t values by the ratio of a full primary dentition to the total number of erupted teeth examined, thereby providing a more representative measure of caries involvement at the population level. This proposed index is intended for cross-sectional interpretation at a specific time point and should be interpreted in conjunction with the conventional dmf-t index. The method for calculating the modified index is illustrated in Figure 2. The modified dmf-t index (mdmf-t) represents a proposed measure that has not yet been formally validated; accordingly, it was used solely for descriptive comparison between study phases and excluded from inferential statistical analyses.

The parent questionnaire is presented in Table 2. The questionnaire was used to collect information on child demographics, early feeding practices, exposure to oral health-related information during pregnancy, and parental educational background. The questions addressed infant feeding during the first six months of life, types and methods of fluid intake, and whether parents received oral health advice for themselves or their child during pregnancy. Parental awareness of the importance of brushing primary teeth was assessed at an attitudinal level only. In addition, the highest level of education attained by both mothers and fathers was recorded. Statistical analyses were conducted to examine associations between children’s dental status and parental responses. The questionnaire was not formally psychometrically validated, as it was developed for descriptive and comparative use in nursery-based epidemiological surveys rather than for scale construction. Its repeated use in both study phases ensured internal consistency and comparability over time. The questionnaire was not designed to assess daily oral hygiene behaviors (e.g., toothbrushing frequency and fluoride use), as the study focus was feeding-related practices and prenatal information rather than home oral hygiene routines. Further questions, such as those concerning toothbrushing practices and fluoride use, were omitted due to the high likelihood of reporting bias associated with self-reported behaviors. The included questions were primarily situational rather than behavioral, as factors such as breastfeeding versus formula feeding are often determined by circumstances rather than parental preference.

We conducted a statistical comparison of the data from 2019 and 2024, focusing on caries prevalence, caries intensity (measured by the experience of decayed, missing, and filled teeth—dmf-t), SiC, and shifts in parental attitudes.

### Statistical Methods and Analysis

Normality of continuous variables was assessed using the Shapiro–Wilk test and visual inspection of boxplots. The boxplots showed multiple outliers and marked skewness in several variables, confirming the nonnormal distribution of the dataset. Therefore, between-group comparisons for continuous variables were performed using nonparametric methods (Kruskal–Wallis and Mann–Whitney U tests). The Mann–Whitney U test was employed to assess differences in the number of carious teeth between children whose parents/guardians received advice on dental prevention and those whose parents did not. Categorical variables were analyzed using the chi-square test. The chi-square test was applied to evaluate differences in the sugar content of fluids consumed regularly by children, based on parental awareness. The examined group was classified into two categories based on the type of drinking vessel used by the child: one group, considered more favorable from a dental perspective, does not use a baby bottle, while the other group does. The relationship between feeding methods during the first six months and subsequent fluid intake habits was also assessed. The Kruskal–Wallis test examined the correlation between parents’ highest level of education and the prevalence of dental caries. For comparison of the data from 2019 and 2024, we applied the Pearson chi-square test (prevalence and intensity) and the Mann–Whitney test (SiC).

Multivariable logistic regression analyses were performed to assess the independent associations between parental educational level, early feeding practices, beverage consumption characteristics, and the presence of dental caries. Data collection was limited to selected variables relevant to the study objectives, which may limit interpretability. All analyses were performed using SPSS Statistics version 24. Results with a *p*-value of <0.05 were considered statistically significant. Because this study aimed to capture real-world nursery populations rather than test a single primary hypothesis, post hoc power calculations were not performed.

## 3. Results

### 3.1. Dental Screening

A total of 636 toddlers participated in our dental screening, 362 in 2019, and 274 in 2024 (Table 3). Caries prevalence decreased from 15.46% in 2019 to 13.87% in 2024. In 2019, 6045 teeth were examined, with 248 (4.01%) affected by caries. The highest dmf-t index was 20. In 6%, S-ECC reached such a severe stage that the lower anterior teeth were also cavitated (S-ECC III). In the study population, no treated teeth were found, i.e., filled (RI = 0%) or removed due to caries [6].

**Figure 3:**
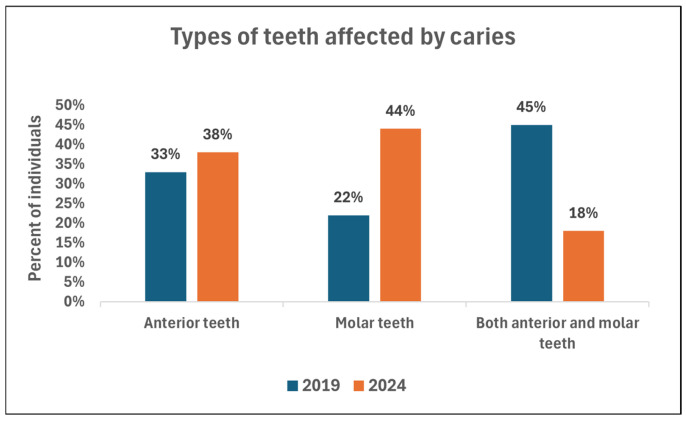
Types of primary teeth affected by caries in the 2019 and 2024 study.

Five years later, 4863 teeth were examined, with 105 affected by caries (2.15%). No filled tooth was found during examinations (RI = 0%). However, earlier, in one case, four upper incisors were extracted due to caries under general anesthesia. Localization of caries has slightly changed over the years (Figure 3). More anterior teeth were involved recently (38% vs. 33%), with an increase in solely molar involvement (44% vs. 22%). Decline is apparent in the co-involvement of anterior and molar teeth (18% vs. 45%).

The comparison of the two phases (2019 vs. 2024) showed a decrease in caries prevalence (15.46% and 13.87% respectively), although it was not significant (*p* = 0.470). However, caries intensity significantly decreased (*p* = 0.025), and a strong tendency could be observed with near statistical significance regarding the SiC index (*p* = 0.052; 2019 mean: 2.09, med: 1.0; 2024 mean: 1.12, med: 0).

### 3.2. Parent Questionnaire

**Figure 4:**
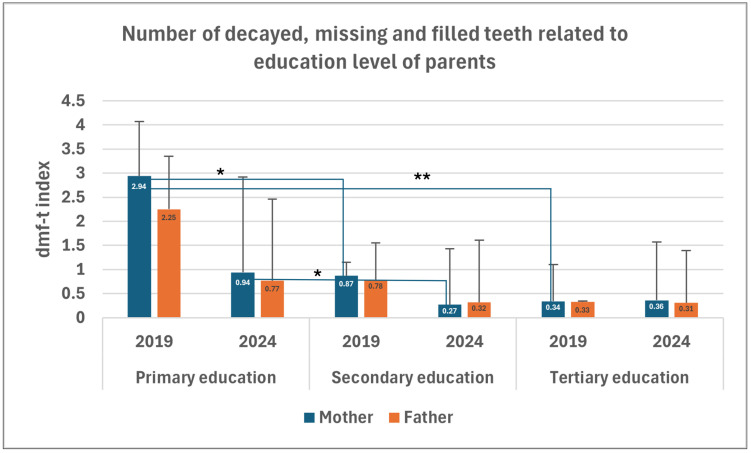
Caries experience of children in 2019 and 2024, measured by the dmf-t index, in relation to parental education level. (dmf-t: number of decayed, missing, and filled teeth; * *p* < 0.05; ** *p* < 0.01).

Five hundred–seventy parents completed and returned the questionnaire on the two occasions. Parental education: In the first phase of the study (2019), mothers’ education level was significantly negatively correlated with caries prevalence (*p* = 0.043). Children of mothers with primary education showed a higher prevalence of dental caries (37.5%) than those whose mothers had secondary (16.1%) or higher education (13.6%) (Table 4). Regarding caries intensity (dmf-t), the difference between higher and secondary education was not significant (*p* = 0.470). However, significant differences were observed in all other comparisons: primary vs. tertiary education (*p* = 0.004) and primary vs. secondary education (*p* = 0.012) (Figure 4). Caries intensity was almost 3.5 times higher among children of mothers with primary education compared to the secondary education group, and about nine times higher than that in the tertiary education group. Only a statistically suggestive trend was detected between fathers’ education and their children’s caries intensity (*p* = 0.088), and no correlation with caries prevalence (Table 4).

In the second phase (2024), neither maternal nor paternal highest education showed correlation with caries prevalence in children (*p* = 0.145 and *p* = 0.136 respectively). With respect to caries intensity, a significant difference was observed between children of mothers with primary and secondary education (*p* = 0.042), while the difference between those with primary and tertiary education was only weakly significant in 2024 (*p* = 0.073) (Figure 4).

Prenatal information on oral health: A higher proportion of parents received oral health advice regarding their child in 2019 than in 2024 (49% vs. 33.7%). This advice was not significantly associated with the number of carious teeth in either year (2019: *p* = 0.196; 2024: *p* = 0.803) and showed only weak significance for health-conscious beverage choices in 2019 (*p* = 0.062), with no association in 2024 (*p* = 0.249).

Nutrition: Although consumption of cariogenic beverages decreased from 75.4% in 2019 to 69.4% in 2024, the difference was only suggestive (*p* = 0.092). In terms of the type of drinking vessel (baby bottle vs. cup) and its association with the prevalence of ECC, our previous study indicated a weak, statistically significant trend, suggesting a potential protective effect of drinking from cups (*p* = 0.069). However, this association was not observed in more recent research, where the relationship was not statistically significant (*p* = 0.448).

Evaluation of correlations among variables: Parental education was significantly associated with children’s drinking practices. In 2019, higher maternal education was associated with increased cup use, with a significant decline observed at lower education levels (*p* = 0.009). No significant association was found for paternal education (*p* = 0.199). In 2024, a significant correlation between cup use and education level was observed for both mothers (*p* = 0.005) and fathers (*p* = 0.001) (Table 4).

At the 2019 screening, higher parental education was associated with a higher proportion of water consumption and a lower intake of sugary beverages. This association was statistically significant for maternal education (*p* = 0.028) and statistically suggestive for paternal education (*p* = 0.05). At the 2024 screening, no statistically significant association was detected; however, a positive trend persisted for both parents (mothers *p* = 0.801; fathers *p* = 0.699) (Table 4).

In both study phases, mothers with higher education breastfed their children during the first six months at significantly higher rates (2019: *p* = 0.006; 2024: *p* = 0.002) (Table 4). Other sociological or socioeconomic factors were not assessed. We have no information on whether the parents raise their children alone or together.

In both phases, initial feeding practices were strongly associated with subsequent drinking modality. Bottle use was significantly more frequent among children who were formula-fed or mixed-fed compared with those exclusively breastfed during the first six months (*p* < 0.001) (Table 5).

In the 2019 survey, where water was the most commonly offered beverage, 52.7% of children had been breastfed, 14.9% formula-fed, and 32.4% mixed-fed, with no statistically significant association observed (*p* = 0.316). In 2024, a statistically suggestive trend was identified, with corresponding proportions of 54.6%, 21.6%, and 23.8%, respectively (*p* = 0.054) (Table 6).

In 2019, parents who had received oral health information were more likely to offer drinks from a cup than from a baby bottle (47.7% vs. 42.8%), although this difference was not statistically significant (*p* = 0.453). However, in 2024, the difference reached statistical significance (2024: 38.1% vs. 27.4%, *p* = 0.022)

Multivariable logistic regression analyses were performed separately for the 2019 and 2024 cohorts. In the 2019 cohort, maternal educational level was significantly associated with caries occurrence, with children of mothers with a low educational level showing markedly higher odds of dental caries (OR = 11.17, 95% CI: 1.64–76.37; *p* = 0.014). In addition, consumption of sugar-containing beverages was independently associated with an increased likelihood of dental caries compared with exclusive water consumption (OR ≈ 3, 95% CI: 0.681–4.288, *p* = 0.046). In contrast, in the 2024 cohort, none of the examined variables showed statistically significant independent associations with dental caries, although the direction of these odds ratios was similar to that observed in 2019 (OR ≈ 1.3, OR ≈ 1.3 respectively), and the confidence intervals were wide (CI = 0.143–4.006, CI ≈ 0.325–1.949 respectively) and included unity for all predictors.

## 4. Discussion

Populations at the highest risk of ECC are often underrepresented in research studies. In Hungary (Pécs), no studies had been published on the dental screening of children under 36 months prior to our 2019 investigation. In this study, we assessed the dental status of toddlers and examined parental behaviors related to feeding practices, parental educational level, and oral health information received during prenatal dental care concerning the child’s future oral health [6]. The examined population is largely overlooked by the dental care system, despite the fact that definitive dental treatment in this age group is often challenging and, in many cases, can only be performed under general anesthesia, which is commonly associated with postoperative complications [2,22]. These circumstances underscore the critical importance of early prevention.

Following the outbreak of COVID-19, lockdown measures substantially altered daily life. Routine screenings were suspended, and access to both dental and general healthcare services became extremely limited in order to reduce viral transmission [23]. Consequently, many expectant mothers may have missed essential dental screenings [24], which could otherwise have provided valuable guidance regarding infant oral health. However, as demonstrated by our 2019 findings, receipt of such information was not universal even before the pandemic. Although we initially expected a deterioration in epidemiological outcomes, our follow-up study revealed improvement in the caries intensity of children born during the pandemic period. Evidence from the literature suggests that pandemic-related changes in daily routines may have influenced oral health behaviors in complex and heterogeneous ways. Liu et al. reported increased oral hygiene routines among children [25], whereas Palaz et al. observed no significant effect of the pandemic on oral hygiene behaviors in children with high caries risk [26]. In contrast, Wdowiak-Szymanik and Grocholewicz found that participants recognized negative changes in oral health behaviors and, following the pandemic, expressed greater appreciation for the importance of regular dental visits and preventive oral healthcare [27]. Dietary habits also changed during the pandemic. While several studies reported increased overeating and snacking, Titis observed a tendency toward healthier food choices [28]. Collectively, these behavioral adaptations may partially explain the outcomes observed in the present study.

In our recent examination, caries prevalence was 13.87%, compared with 15.46% in 2019 (*p* = 0.470). This prevalence is considerably lower than the global ECC prevalence of 49% reported by Maklennan et al., with a European estimate of 42% [29]. This less favorable outcome is likely due to the wider examined age range (1–6 years), as found in the definition of ECC. Within the age range examined in our study, caries prevalence reported by other authors varies widely, from 1% to 37.5%, regardless of geographic region [30]. The lower caries prevalence observed in our study may also be partly attributable to underdiagnosis, given the circumstances of the clinical examinations and the data-recording protocol. Specifically, only ICDAS scores 3–6 were included, potentially resulting in the omission of early non-cavitated lesions and underestimation of true disease burden.

**Figure 5:**
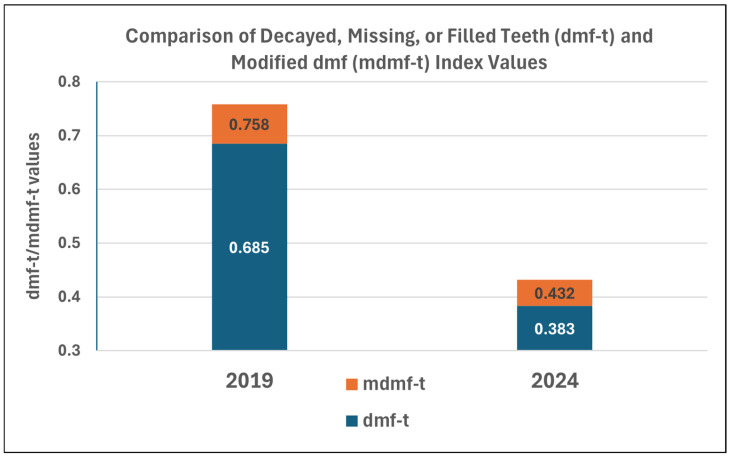
The difference between the dmf index values and the mdmf-t index values. (dmf: number of decayed, missing due to caries, and filled teeth; mdmf: modified dmf).

A significant difference was observed in caries experience, as reflected by higher dmf-t values in 2019 (pre-pandemic) compared with 2024 (COVID-era) (*p* = 0.025). This finding indicates that although the proportion of affected children did not decrease significantly, the number of affected teeth per child declined. As noted by Dye et al., the number of erupted teeth complicates the interpretation of the results in the literature [31]. This supports the use of indices that account for eruption status, such as the proposed mdmf-t index, which was applied in the present study to reflect individual caries experience (Figure 5). Regarding the SiC index, a statistically suggestive trend toward decreasing dmf-t values was observed (*p* = 0.052). A statistically suggestive decrease was also observed in the SiC index (*p* = 0.052). Contrary to our initial hypothesis, reduced access to healthcare during the pandemic did not result in increased caries prevalence or severity among children born during the COVID-19 period.

A meta-regression and systematic review by Kassebaum et al. identified untreated caries in primary teeth as the tenth most prevalent condition globally, affecting approximately 621 million individuals [4]. This is consistent with our findings, as zero RI values were recorded in both study phases. This may be attributable to limited dental screening and delayed diagnosis, reducing the likelihood of non-invasive treatment options in this age group. Consequently, rapidly progressing undiagnosed lesions often require invasive interventions, frequently performed under general anesthesia in specialized care settings. These findings further emphasize the importance of preventive strategies.

Several factors potentially influencing oral health were also examined, including parental education, exposure to oral health information, and behavioral practices such as infant feeding, fluid consumption, and drinking modality (types of vessels used for feeding and drinking).

In 2019, maternal education significantly correlated with the oral health of children, influencing both caries prevalence and caries intensity. This finding aligns with a Portuguese study, which reported that 90.9% of children were caries-free when their parents—especially mothers—had a higher level of education [31]. However, in our 2024 study, no significant correlation was observed with caries prevalence, and the association with caries intensity was also weaker. This suggests that the COVID-19 pandemic may have attenuated the relationship between educational attainment and oral health outcomes. Interestingly, a recent Italian study reported higher caries experience among children of highly educated mothers, potentially related to increased workload and reduced supervision of daily oral hygiene practices [32]. A similar mechanism may partly explain our observations.

In Hungary, professional guidance on infant oral health is primarily limited to mandatory preventive dental care during pregnancy. Children typically do not attend dental consultations before the age of three, leaving pediatricians and district nurses as the main providers of preventive advice and early detection. In 2019, only 49% of mothers reported receiving oral health or dietary counseling aimed at ECC prevention. As anticipated, this proportion declined further in 2024, to 33.7%, likely due to reduced availability and utilization of prenatal dental services during COVID-19 lockdowns. The pandemic led to significant disruptions in prenatal care services, including dental care. Studies have reported a marked decrease in dental service utilization during lockdown periods, particularly for routine and preventive services. This reduction in access likely contributed to fewer opportunities for pregnant women to receive dental counseling and preventive advice, which are factors that may have downstream effects on their children’s oral health [33,34,35]. Furthermore, our findings indicate that parental oral health education during pregnancy—at least in its current form—is inadequate. Prenatal awareness of oral health remains insufficient, and the methods of delivering health care advice should evolve to align with contemporary norms. A systematic review by Farrokhi et al. supports the notion that incorporating modern communication channels, such as social media and short educational videos, can enhance engagement and improve knowledge retention among expectant parents [36].

Parental education emerged as a key determinant of drinking modality. Parents with secondary or higher education were more likely to offer drinks from a cup rather than a baby bottle, consistent with findings by De La Hunty et al. [37]. In contrast, a Chinese study reported a negative association only with paternal education [38]. In our results, a positive trend was observed between higher parental education levels and reduced consumption of cariogenic beverages in children. Similar associations have been reported previously, with children of less-educated parents consuming sugary drinks more frequently, likely reflecting differences in health literacy, dietary habits, and home environments [39,40]. Overall, a decreasing trend in cariogenic beverage consumption was observed across study periods, potentially contributing to improved caries intensity outcomes. This contrasts with reports of more cariogenic dietary patterns during lockdowns, characterized by increased sugar intake and eating frequency [41,42].

Both study phases demonstrated a strong association between initial feeding practices and later drinking modality (*p* < 0.001). Mothers who exclusively breastfed during the first six months, in accordance with WHO recommendations, were more likely to offer drinks from a cup and avoid baby bottle use. These findings highlight the long-term benefits of exclusive breastfeeding. In addition to its well-established developmental advantages, breastfeeding supports optimal orofacial development, and cup feeding has been shown to promote more favorable muscle activity compared with bottle feeding [43,44].

The present study provides a cohort-specific evaluation of selected factors associated with dental caries in young children. While the multivariable analysis of the 2019 dataset identified maternal educational level and beverage consumption as significant independent factors, these associations were not reproduced in the 2024 cohort. The absence of statistically significant associations in the later dataset does not necessarily contradict the earlier findings but may reflect temporal changes in parental awareness, preventive practices, dietary habits, or access to oral health information. In addition, a more homogeneous distribution of characteristics in the 2024 cohort may have attenuated detectable effect sizes in the multivariable model.

Based on the findings of the present study, our initial hypothesis was rejected, as children born during the COVID-19 pandemic demonstrated improved caries intensity outcomes. Evidence from a recent cross-cultural, cross-sectional study indicates that the pandemic was associated with increased general health awareness in some populations [16], potentially reflecting heightened perception of health vulnerability during periods of restricted access to routine healthcare. While this may have contributed to increased attention to preventive behaviors, including those relevant to oral health, this interpretation remains speculative, and causality cannot be established. Given conflicting evidence in the literature, further investigation is warranted [45,46,47,48].

This study was not designed as a comprehensive assessment of all behavioral risk factors for early childhood caries. In particular, daily oral hygiene practices were intentionally excluded from the questionnaire. Self-reported toothbrushing behaviors in very young children are known to be highly susceptible to social desirability and recall bias, especially when reported by parents. Therefore, the questionnaire focused on feeding-related practices and prenatal information, which are more situational in nature and less influenced by subjective reporting [49].

Limitations: Caries diagnosis in the present study was limited to a dichotomous classification of cavitated caries (ICDAS ≥ 3) versus teeth with ICDAS ≤ 2. Because lesion severity was not evaluated separately and a single diagnostic threshold was applied consistently across examiners and study phases, the potential impact of inter-examiner variability is reduced; however, the absence of formal inter- and intra-examiner reliability statistics remains a methodological limitation. The exclusion of ICDAS scores 1 and 2 may have resulted in the underestimation of early caries lesions and overall prevalence. However, the validity of including non-cavitated lesions in epidemiological surveys remains debated and has not been adequately evaluated, as highlighted by Mendes et al. [50], supporting the use of higher ICDAS thresholds in population-based studies.

Dental screenings were conducted exclusively in nurseries in Pécs, a university city that may exhibit more favorable oral health profiles than the national average. Children not attending nurseries, who may differ in socioeconomic background and caries risk, were not represented. Participation was further limited to children whose parents provided written informed consent, and no data were available on non-participants or refusal rates. In addition, absences on screening days reduced the final sample size. Together, these factors may have introduced selection bias, potentially limiting the generalizability of the findings to the broader population of children under three years of age. Furthermore, the relatively small number of children whose parents had only primary education represents an additional limitation and may be related to differences in parental employment patterns, with parents of lower educational attainment more likely to remain at home with their children for longer periods.

Because dental screenings were conducted prospectively in two distinct time periods, the examiners were not blinded to the study phase, which may represent a source of observer bias; however, standardized protocols and consistent diagnostic thresholds were used across both phases, with consistent recruitment strategies and examination protocols, allowing for meaningful longitudinal comparisons.

The questionnaire was not subjected to formal psychometric validation. Although its repeated use across study phases ensured internal consistency and comparability over time, the absence of validation may limit construct validity.

## 5. Conclusions

Although caries intensity outcomes improved among children born during the COVID-19 era, ECC prevalence remains substantial. The initial null hypothesis, that the pandemic would adversely affect children’s oral health, was not supported. Contrary to expectations, children born during the pandemic demonstrated more favorable oral health outcomes than the pre-pandemic cohort.

Parental oral health education remains limited, as reflected by minimal differences in caries prevalence between informed and uninformed parents. While reduced access to prenatal oral health information was anticipated to negatively influence outcomes, it showed no significant association with caries prevalence and only a statistically suggestive relationship with healthier beverage choices.

These findings underscore the need for comprehensive, standardized ECC prevention guidelines in Hungary. Early and regular oral health screening from 6 to 12 months of age, in line with IAPD recommendations, should be implemented. Integration of preventive dental strategies and basic diagnostic skills into pediatric and nursing care through mandatory training may further enhance early detection and prevention, as infants and toddlers are routinely seen within primary child healthcare services during their early years. Our long-term goal is to establish oral health as a core component of a healthy lifestyle from early childhood and to reduce ECC prevalence through an effective, prevention-oriented network aligned with global standards set by the WHO and IAPD.

## Figures and Tables

**Table 1:** Definition of early childhood caries (ECC) and severe early childhood caries (S-ECC) according to age based on criteria by the American Academy of Pediatric Dentistry [9]. (dmf-s: number of decayed, missing, and filled tooth surfaces).

Age (Months)	Early Childhood Caries (ECC)	Severe Early Childhood Caries (S-ECC)
<12		1 or more cavitated or non-cavitated smooth surface caries
12–23		
24–35		
36–47	1 or more dmf-s	1 or more cavitated, filled, or missing upper anterior primary teeth due to caries, or a dmf-s > 3
48–59	1 or more dmf-s	1 or more cavitated, filled, or missing upper anterior primary teeth due to caries, or a dmf-s > 4
60–71	1 or more dmf-s	1 or more cavitated, filled, or missing upper anterior primary teeth due to caries, or a dmf-s > 5

**Table 2:** Parent questionnaire with dichotomous and open-ended questions. (Also used in prior publication by Radacsi et al. 2021) [6].

	Personal Data:	
	Name, Sex, Date of Birth	
	Questions	Choice of Answers:
Nutrition	feeding in the first 6 months	breastfeeding, formula, breastfeeding and formula
	type of fluid intake (more choices are accepted)	water, tea without sugar, tea with sugar, formula, other: _______
	method of fluid intake	baby bottle, cup, other:_______
Information given	Have you received any information related to your child’s oral health during pregnancy?	yes/no
	If your answer is yes, from whom?	dentist, pediatrician, home visitor, nurse, other: _____
	What information have you received?	textual answer
	Have you received any information related to your own oral health during pregnancy?	yes/no
	If your answer is yes, from whom?	textual answer
	Do you think it is important to brush the primary teeth?	yes/no
Highest level of education	mother	primary, secondary, tertiary
	father	primary, secondary, tertiary

**Table 3:** Results of dental screening in 2019 and 2024 (dmf-t: decayed, missing due to caries, and filled tooth; SiC-index: significant caries index; RI: restorative index).

Results of Dental Screening	2019	2024
Number of examined children	362	274
- Girls	186	138
- Boys	176	136
Number of examined children with caries (caries prevalence)	56 (15.46%)	38 (13.87%)
- Girls	25 (44.64%)	22 (57.89%)
- Boys	31 (55.36%)	16 (42.11%)
Age (months) ± SD	28.49 ± 5.52	29.37 ± 4.56
dmf-t index ± SD	0.685 ± 2.20	0.383 ± 1.29
Number of erupted teeth in average ± SD	18.06 ± 3.13	17.76 ± 3.03
Completely erupted primary dentition *n* (%)	214 (58.9%)	134 (48.9%)
Modified dmf-t index (mdmf-t index) ± SD	0.758 ± 2.42	0.432 ± 1.43
Number of examined teeth	6045	4867
Number of teeth affected by caries (%)	248 (4.01%)	105 (2.15%)
SiC-index ± SD	2.06 ± 3.33	1.15 ± 2.04
Restorative index	0%	0%

**Table 4:** Correlations between the parental highest level of education and prevalence of caries, along with preventive factors, in 2019 and 2024. (*n*: number of observations in the category; * *p* < 0.05; ** *p* < 0.01). In cases where questionnaire data were incomplete, children with missing responses were excluded from the respective analyses; no imputation was performed.

		Level of Education (2019/2024)							
		Primary *n* (%)		Secondary *n* (%)		Tertiary *n* (%)		*p* Value	
		2019	2024	2019	2024	2019	2024	2019	2024
Mother	Child’s caries prevalence	6 (37.5)	5 (29.4)	18 (16.1)	13 (11.8)	24 (13.6)	18 (13.4)	* 0.043	0.145
	Water as preferred fluid	2 (12.5)	4 (23.5)	27 (24.1)	34 (30.9)	46 (26.3)	38 (28.6)	* 0.028	0.801
	Drinking from cup	5 (31.3)	3 (18.8)	45 (41.3)	34 (31.8)	100 (66.7)	46 (34.6)	** 0.009	** 0.005
	Breast feeding	6 (37.5)	5 (37.5)	38 (34.2)	45 (40.9)	94 (53.4)	80 (60.2)	** 0.006	** 0.002
Father	Child’s caries prevalence	4 (25)	7 (25.9)	28 (18.9)	15 (11.6)	16 (11.9)	13 (12.7)	0.164	0.136
	Water as preferred fluid	0 (0)	6 (22.2)	36 (66.7)	39 (30.2)	39 (78)	30 (29.7)	0.050	0.699
	Drinking from cup	7 (43.8)	2 (7.70)	68 (46.3)	46 (36.2)	74 (56.5)	33 (32.7)	0.199	**0.001

**Table 5:** Association between initial feeding pattern and consecutive method of fluid intake in the 2019 and 2024 surveys. (*n*: number of observations in the category). In cases where questionnaire data were incomplete, children with missing responses were excluded from the respective analyses; no imputation was performed.

		Brest Feeding *n* (%)	Formula *n* (%)	Mixed Feeding *n* (%)	*p* Value
Fluid intake with baby bottle (%)	2019	43 (31.9)	37 (71.2)	70 (61.9)	<0.001
	2024	80 (48.5)	48 (87.8)	87 (86.9)	

**Table 6:** The relationship between a child’s initial feeding method and their later fluid consumption in the 2019 and 2024 surveys. The percentage shows the ratio of the initial feeding method for mainly water-consuming children. (*n*: number of observations in the category). In cases where questionnaire data were incomplete, children with missing responses were excluded from the respective analyses; no imputation was performed.

	Initial Feeding/Later Fluid Consumption	Breast Feeding *n* (%)	Formula *n* (%)	Mixed Feeding *n* (%)	*p* Value
Water is main drink (%)	2019	39 (52.7)	11 (14.9)	24 (32.4)	0.316
	2024	53 (54.6)	21 (21.6)	23 (23.8)	0.054

## Data Availability

The original contributions presented in this study are included in the article. Further inquiries can be directed to the corresponding author.

## Abbreviations

The following abbreviations are used in this manuscript:

AAPD	American Academy of Pediatric Dentistry
dmf-t	Decayed, Missing, and Filled Teeth
ECC	Early Childhood Caries
IAPD	International Association of Pediatric Dentistry
ICDAS	International Caries Detection and Assessment System
RI	Restorative Index
SiC	Significant Caries Index
WHO	World Health Organization
